# Do pesticide and pathogen interactions drive wild bee declines?

**DOI:** 10.1016/j.ijppaw.2022.06.001

**Published:** 2022-06-13

**Authors:** Lars Straub, Verena Strobl, Orlando Yañez, Matthias Albrecht, Mark J.F. Brown, Peter Neumann

**Affiliations:** a Institute of Bee Health, Vetsuisse Faculty, University of Bern, Bern, Switzerland; b Agroecology and Environment, Agroscope, Zürich, Switzerland; c Department of Biological Sciences, Royal Holloway University of London, Egham, UK; d Swiss Bee Research Centre, Agroscope, Bern, Switzerland

**Keywords:** Fitness, Interactions, Pesticides, Pathogens, Wild bees

## Abstract

There is clear evidence for wild insect declines globally. Habitat loss, climate change, pests, pathogens and environmental pollution have all been shown to cause detrimental effects on insects. However, interactive effects between these stressors may be the key to understanding reported declines. Here, we review the literature on pesticide and pathogen interactions for wild bees, identify knowledge gaps, and suggest avenues for future research fostering mitigation of the observed declines. The limited studies available suggest that effects of pesticides most likely override effects of pathogens. Bees feeding on flowers and building sheltered nests, are likely less adapted to toxins compared to other insects, which potential susceptibility is enhanced by the reduced number of genes encoding detoxifying enzymes compared with other insect species. However, to date all 10 studies using a fully-crossed design have been conducted in the laboratory on social bees using *Crithidia* spp. or *Nosema* spp., identifying an urgent need to test solitary bees and other pathogens. Similarly, since laboratory studies do not necessarily reflect field conditions, semi-field and field studies are essential if we are to understand these interactions and their potential effects in the real-world. In conclusion, there is a clear need for empirical (semi-)field studies on a range of pesticides, pathogens, and insect species to better understand the pathways and mechanisms underlying their potential interactions, in particular their relevance for insect fitness and population dynamics. Such data are indispensable to drive forward robust modelling of interactive effects in different environmental settings and foster predictive science. This will enable pesticide and pathogen interactions to be put into the context of other stressors more broadly, evaluating their relative importance in driving the observed declines of wild bees and other insects. Ultimately, this will enable the development of more effective mitigation measures to protect bees and the ecosystem services they supply.

## Highlights

• Pesticide-pathogen interactive effects remain poorly understood for wild bees.
• Current data has a strong focus on laboratory and social bee species.
• There is an urgent need to consider other pathogens and solitary bee species.
• An improved understanding of interactions and the mechanistic pathways is key for bee conservation.

## Graphical abstract

**Figure:**
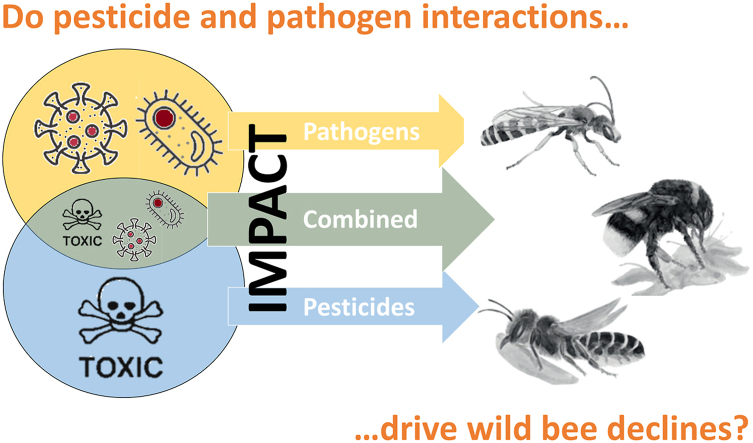

## 1 Introduction

Global declines of the entomofauna are occurring at an alarming rate (Cardoso et al., 2020; Eisenhauer et al., 2019; Hallmann et al., 2017). This is of considerable concern as insects play an indispensable role in terrestrial as well as aquatic environments by providing key ecosystem services (Costanza et al., 1997). A wide array of stressors likely govern the observed insect declines and losses, including habitat destruction (Marshall et al., 2017), pest and pathogens (Neumann and Carreck, 2010; Ravoet et al., 2014), climate change (Soroye et al., 2020), intensified agriculture (Díaz et al., 2019; Winfree, 2010) and environmental pollution (Straub et al., 2020). These stressors, however, most certainly do not act in isolation. Rather they simultaneously interact with one another thereby generating complex effects that may amplify the direct consequences of a single given stressor (Jackson et al., 2016). Insect pollinators are no exemption to such concurrent exposure scenarios (Goulson et al., 2015a; Siviter et al., 2021a; Vanbergen et al., 2013), which are argued to be a core explanation for increasing reports of wild bee declines (Biesmeijer et al., 2006; Potts et al., 2010). However, there are wide gaps of knowledge surrounding these complex interactions and how they may affect wild bee health and populations (Dicks et al., 2021).

Bees are key pollinators of various native plant and crop species, thereby providing immense ecosystem services and sustaining human food security (Diaz et al., 2019; Garibaldi et al., 2016; Potts et al., 2010). With roughly 20′400 described species (Engel et al., 2020; Michener, 2000), bees are a highly diverse group of pollinators, encompassing a range of morphological differences, nesting behaviours, life-histories (e.g., solitary vs. eusocial), phenologies, and foraging habits (e.g., specialists vs. generalists) (Michener, 2000). However, various bee species are currently considered to be critically endangered (Nieto et al., 2014; Zattara and Aizen, 2021), including solitary as well as social bee species (Biesmeijer et al., 2006; Cameron et al., 2011; Powney et al., 2019). Recent research efforts focused on understanding the relationship between environmental stressors and reported wild bee declines have implicated intensive agricultural management practices as being a major driver (Baude et al., 2016; Hayes and Hansen, 2017; Potts et al., 2016; Sánchez-Bayo and Wyckhuys, 2019)). Indeed, large-scale intensified agriculture has reshaped our natural landscapes (Kremen et al., 2002; Tilman et al., 2002), which has led to the reduction of foraging and nesting sites, as well as increased agrochemical exposure for bees and other wild animals (Mancini et al., 2019; Neumann et al., 2015; Woodcock et al., 2016b). A further factor argued to influence population dynamics of wild bees are parasites and pathogens (Cameron et al., 2011; Meeus et al., 2011; Tsvetkov et al., 2021). Undeniably, a plethora of laboratory studies have revealed negative impacts of pesticides and pathogens individually on bee survival, behaviour, physiology, and reproduction (e.g., Blacquière et al., 2012; Schmid-Hempel, 1998). Yet, while the single effects of common bee pathogens (e.g., viruses or *Nosema* spp. parasites) as well as of frequently used pesticides (e.g., neonicotinoid insecticides) have been comparatively well studied (e.g., Grupe and Alisha Quandt, 2020; Pisa et al., 2014; Siviter et al., 2021b, 2021a; Tehel et al., 2016), their interactive effects are poorly understood (Collison et al., 2016). Furthermore, it remains unclear whether a direct link between the exposure to these two groups of stressors and recent observed field declines is even present. Indeed, while some field data are available suggesting the impact of agrochemicals on wild bees in the field (Powney et al., 2019; Rundlöf et al., 2015), there is a lack in knowledge on the actual role of any pathogen on wild bee populations. One exception seems to be the impact of the ectoparasitic mite *Varroa destructor* and associated viruses (Neumann et al., 2012) on wild and feral honey bees in Europe and North America (Kraus and Page, 1995). The latter is an important point as honey bees (*Apis* spp.) consist of at least 11 species (Otis, 2019) and only two species are managed (*Apis mellifera* and *Apis cerana*). Even for these two managed honey bees species, the vast majority of colonies in Africa and Asia are still wild rather than managed (Hepburn and Radloff, 1998; Michener, 2000; Otis, 2019). Here, we review the literature on the impact of pesticide and pathogen interactions on wild bees. Based on the current evidence we put this specific interaction scenario into context with other stressors and evaluate their relative importance for recent global wild bee declines. Furthermore, we identify current knowledge gaps and suggest avenues for the future research required if we aim to effectively mitigate the role of pesticide and pathogen interactions in the ongoing decline of wild bees.

## 2 Methods

All bee species were considered for the literature review. However, studies using managed honey bees (*Apis mellifera*) were excluded as numerous previous reviews have focused on this species to the exclusion of other bees (e.g., Bird et al., 2021; Collison et al., 2016; Siviter et al., 2021b). We used Web of Science as our search engine, using the databases ‘Web of Science Core Collection’ (1990 to present) and ‘BIOSIS Citation Index’ (2006 to present). The search terms used were based on three groups: (1) Family or study organism (e.g., Andrenidae or solitary bee); (2) environmental stressors (i.e., parasite/pathogen or pesticide); and (3) response variable (e.g., survival or sperm). The full search terms used can be found in the supplementary information.

The literature search was conducted on November 18, 2021 and yielded 6′458 papers. Articles that did not include data (e.g., reviews, comments, opinions, or editorials), as well as irrelevant studies (e.g., 'rheumatology'), were excluded. Thereafter, 5′069 publications remained. We screened the titles of all papers and excluded papers that did not mention one of the potential environmental stressors as well as papers that used managed honey bees as the only model organism. In total, 3′576 titles were excluded, leaving 1′493 papers. The abstracts were then screened to determine whether (1) the study included combined stressor exposure (i.e., pesticide and parasites or pathogens), and (2) measured a response variable relating to bee health (e.g., survival, physiology, fitness). A further 1′460 were excluded at this stage, leaving a total of 33 papers. The remaining papers were carefully read by one researcher (L.S., V.S., or O.Y.) to determine if the study followed the inclusion criteria. For a study to be included, it had to satisfy the following inclusion criteria: (1) the publication had to address the effect of a combination of parasite/pathogen/pest and agrochemical (i.e., herbicides, fungicides, insecticides, acaricides, miticides, biocides, etc.) on wild bee health; and (2) the experimental design had to be fully crossed (i.e., control, treatment stressor 1, treatment stressor 2, and treatment stressor 1 & 2). All studies of individual bees, bees grouped in cages, or colonies at any life stage were included and all measured response variables were considered. Studies were included even if the interaction between stressors was not explicitly tested or stated. This ultimately led to a total of 10 publications, which were considered within this review. We cross-checked our search with Google Scholar by using the same terms as described above. However, no additional studies were revealed, thus confirming our search in Web of Science was sufficient.

## 3 Results

**Table 1:** Overview of the literature meeting our criteria assessing individual and combined pesticide and pathogen exposure on wild bees.

Authors	Model organism (*species*)	Pesticide(s)	Chemical(s)	Pathogen(s)	Life-stage(s) exposed	Experiment settings	Assessed parameters	Interaction type(s)	Key findings
Fauser-Misslin et al. (2014)	Bumble bee (*Bombus terrestris*)	Insecticide	Clothianidin, thiametoxam	*Crithidia bombi*	Colony	Laboratory	Survival of mother queens	N.A.	Chronic dietary exposure lead to negative effects on worker production, reduced worker longevity and decreased overall colony reproductive success. Further, the authors revealed a significant interaction between neonicotinoid exposure and parasite infection on mother queen survival. Under combined pressure of parasite infection and neonicotinoid exposure, mother queen survival was lowest
Baron et al. (2014)	Bumble bee (*Bombus terrestris*)	Insecticide	lambda (λ)-cyhalothrin	*Crithidia bombi*	Colony and individual workers	Laboratory	Mortality, colony development, reproductive output and body size	None	No significant impact on the susceptibility of workers to *C. bombi* or intensity of parasitic infection; no impact on survival in workers and males.
Baron et al. (2017)	Bumble bee (*Bombus terrestris*)	Insecticide	Thiamethoxam	*Crithidia bombi*	Colony and individual queens	Laboratory	Mortality, colony founding, body mass	None	Exposure to thiamethoxam caused a 26% reduction in the proportion of queens that laid eggs, and advanced the timing of colony initation, yet no effects were observed on the ability of queens to produce adult offspring. No interactive effects were observed between parasite and pesticide.
Fauser et al. (2017)	Bumble bee (*Bombus terrestris*)	Insecticide	Thiamethoxam and clothianidin	*Crithidia bombi*	Queens	Laboratory	Hibernation survival and hibernation weight change of queens	None	Both reduced hibernation success individually, but no additive or synergistic effects were found
Botías et al., 2020	Bumble bee (*Bombus terrestris*)	Insecticide and fungicide	Thiamethoxam, cypermethrin and tebuconazole	*Nosema ceranae*	Colony	Laboratory exposure; evaluation of effects in the field (colonies)	Prevalence of *N. ceranae*, expression levels of immunity and detoxification-related genes, food collection, weight gain, worker and male numbers, and production of worker brood and reproductives	Synergistic and antagonistic	Exposure to pesticide mixtures reduced food collection by bumble bees. All immune related genes were up-regulated in the bumble bees inoculated with *N. ceranae* when they had not been exposed to pesticide mixtures, and bumble bees exposed to the fungicide and the pyrethroid were less likely to have *N. ceranae*. Combined exposure to the three-pesticide mixture and *N. ceranae* reduced bumble bee colony growth, and all treatments had detrimental effects on brood production. The groups exposed to the neonicotinoid insecticide produced 40%–76% fewer queens than control colonies.
Guimarães-Cestaro et al., 2020	Stingless bees (*Tetragonula elegante*)	Herbicide	Glyphosate	Six different viruses (DWV, ABPV, BQCV, KBV, IAPV, and CBPV) and microsporidia (*Nosmema apis* and *Nosema ceranae*)	Individual bees	Bee collection in the field, molecular analysis in the laboratory	Six different viruses (DWV, ABPV, BQCV, KBV, IAPV, and CBPV), microsporidia (*N. apis* and *N. ceranae*), and pesticide residues	N.A.	40–55% of samples had *N. ceranae* but not in the midgut. 23.4% of samples were positive for viruses. ABPV was the most prevalent, followed by DWV and BQCV. All samples of the *T. elongate* showed <0.05 mg/kg glyphosate and its aminomethylphosphonic acid (AMPA) metabolites that is the minimum detection limit, whereas for the other pesticides analyzed were not detected. Due to this low pesticide occurrence, the authors could not evaluate the interaction between pesticide exposure and pathogens in the stingless bees.
Macías-;Macías et al., (2020)	Stingless bee (*Melipona colimana*)	Insecticide	Thiamethoxam	*Nosema ceranae*	Newly emerged bees	Laboratory	Survivorship and cellular immunity (hemocyte concentration)	Synergistic	*N. ceranae* did not affect survivorship. Thiamethoxam at a sublethal concentration reduced the survival. Lowest survivability was for the bees treated with both stressors, which suggests a detrimental synergistic effect due to the interaction of *N. ceranae* and thiamethoxam on the lifespan of *M. colimana*. Bees treated with *N. ceranae* only had significantly lower concentrations of hemocytes in the hemolymph than bees of the rest of the treatments. *N. ceranae* may infect and replicate in stingless bees in the Americas and it may inhibit cellular immunity. Thiamethoxam seems to restrain the replication of *N. ceranae* but may be toxic to *M. colimana* bees at sublethal concentrations, particularly in combination with *N. ceranae* infections, which could have negative implications on their populations and pollination services.
Siviter et al., 2020	Bumble bee (*Bombus terrestris*)	Insecticide	Sulfoxaflor	*Nosema bombi*	Larvae	Laboratory	Mortality, larval growth	Additive and antagonistic	We found no significant impact of sulfoxaflor (5 ppb) or *N. bombi* exposure (50 000 spores) on larval mortality when tested in isolation but found an additive, negative effect when larvae received both stressors in combination. Individually, sulfoxaflor and *N. bombi* exposure each impaired larval growth, although the impact of combined exposure fell significantly short of the predicted sum of the individual effects (i.e. they interacted antagonistically).
Calhoun et al., 2021	Bumble bee	Fungicide	Chlorothalonil	*Nosema bombi*	Worker-produced microcolonies	Laboratory	Microcolony development and production. Produced males were assessed for body size, protein amounts, total infection intensity, extracellular spore loads and survival	None	Development, size, survival and protein amounts of males from microcolonies were not significantly negatively affected by *Nosemabombi* exposure or infection, chlorothalonil exposure, nor their interaction. Additionally, the prevalence and infection intensities at 5 days post-eclosion did not differ. Bees from microcolonies exposed to chlorothalonil exhibited increased spore loads, with spores representing a greater proportion of the total infection intensity. This indicates that in bumble bees, chlorothalonil exposure can interact with *N. bombi* infection to influence a parameter important for transmission dynamics that could affect colony, population or community health.
Straw and Brown, 2021b	Bumble bee (*Bombus terrestris*)	Herbicide	Glyphosate	*Crithidia bombi*	Microcolonies	Laboratory	Mortality, *C. bombi* concentration, and worker reproduction	None	Authors found no effects of acute or chronic exposure to glyphosate, over a range of timespans post-exposure, on mortality or a range of sublethal metrics. Further, they found no interaction between glyphosate and *C. bombi* in any metric, although there was conflicting evidence of increased parasite intensity after an acute exposure to glyphosate.

## 4 Discussion

Here, we show that only a limited number of publications have so far addressed the interactive effects of pesticides and pathogens on wild bees (see Table 1). All studies were performed under laboratory conditions using social species (i.e., bumble bees and stingless bees) and exclusively focussed on interactions between insecticides (mainly neonicotinoids) and either *Crithidia* or *Nosema* spp. Whilst often no significant interaction was observed, some studies found evidence for interactions ranging from antagonism to synergism depending on the measured variable. The limited data so far suggest that effects of pesticides most likely override effects of pathogens, probably because bees, feeding on flowers and building sheltered nests, are less adapted to toxins compared to other insects (but see e.g., Tiedeken et al., 2016). There is an evident need to (i) test pesticide and pathogen interactions across a wider range of bee species, (ii) consider other pathogens, (iii) conduct semi-field and field studies, and (iv) focus on measuring impacts on fitness or fitness-relevant traits when assessing these concurrent exposure scenarios. An improved understanding of the mechanistic pathways and consequences of pesticide and pathogen interactions is essential for adequate conservation to mitigate the ongoing global decline of wild bee species.

In contrast to wild bees, studies on interactions between agrochemicals and pathogens/parasites using managed Western honey bees, *A. mellifera*, are far more common (see reviews by Bird et al., 2021; Collison et al., 2016; O'Neal et al., 2018; Sánchez-Bayo et al., 2016; Siviter et al., 2021a). This is likely due to their economic relevance for pollinating agricultural crops as well as wild plants (Calderone, 2012; Hung et al., 2018; Potts et al., 2010), their use, until recently, as the single model bee species for risk assessments of pesticides, but also because their biology is well known and they are easily maintained under both laboratory (Carreck et al., 2020; Williams et al., 2013) and field conditions (Crane, 2009). In brief, findings of both agrochemical exposure and pathogen infection on managed honey bee health vary among studies, making it difficult to draw general robust conclusions on interactive effects of these two stressors (Collison et al., 2016), although a recent meta-analysis concluded that effects are likely additive overall across bees (Siviter et al., 2021a). The variation in findings may be explained by varying exposure and infection regimes, differences in the developmental stages of the insects (e.g., larvae vs adults), inherent variability (e.g., genetics or seasonal variability in pathogen loads) and/or variation amongst studies in methodological approaches. The last issue calling for standardized approaches to investigate managed honey bee health using similar methods (Carreck et al., 2020). In a recent metal-analysis from 26 studies testing combined effects of parasites and pesticides on managed honey bee health, the authors concluded that the combined pesticide-pathogen treatments often revealed antagonistic effects, rather than predicted additive or multiplicative effects (Bird et al., 2021; but see Siviter et al., 2021a). The physiological and genetic mechanisms underlying these antagonistic interactions remain unclear and additional research is needed. Furthermore, the majority of managed honey bee studies focus on *Nosema* spp., *Varroa destructor*, and various bee viruses (e.g., Annoscia et al., 2020; Aufauvre et al., 2012; Coulon et al., 2020; Di Prisco et al., 2013; Harwood and Dolezal, 2020; Odemer et al., 2018; Retschnig et al., 2015; Straub et al., 2019). While the role of *Nosema* spp. for colony health remains controversial, i.e. colonies surviving winter have higher *Nosema* spp. loads (Dainat et al., 2012), there is general consensus that the ectoparasitic mite *V. destructor* and associated viruses currently represent the greatest threat to managed honey bee health (Neumann and Carreck, 2010; Rosenkranz et al., 2010). Of particular concern are the negative impacts of these parasites on host immune competence in honey bees (Di Prisco et al., 2013, 2016). Such negative impacts on immune barriers can then be further exacerbated by concurrent pesticide exposure. For instance, pesticide exposure in honey bees has been shown to interfere with individual immune response by impairing the NF-kB immune signalling pathways, as well as reducing antimicrobial capacity, delaying wound healing and lowering the number of circulating haemocytes (Brandt et al., 2017; Di Prisco et al., 2013; James and Xu, 2012), thus favouring the spread of pathogens and parasites (Annoscia et al., 2020; Di Prisco et al., 2013).

Yet, despite significant advances in identifying interactions at the individual level of managed honey bees, few data exist as to why many of the interactions observed fail to translate into quantifiable effects at the colony level (O'Neal et al., 2018; Osterman et al., 2019). This most likely is due to the ability of honey bees and other social insect species to buffer negative impacts at the colony level (i.e., “superorganism resilience” (Straub et al., 2015)). Moreover, laboratory findings do not necessarily translate into quantifiable effects in the field (Retschnig et al., 2015). Indeed, pesticide exposure and pathogen infection have not yet been found to interact and affect managed honey bee worker survival under field-realistic scenarios (Collison et al., 2016). Whilst consequences of pesticide effects on *Nosema*spp. Infection levels, viral titres, or individual immunity have been observed under controlled laboratory conditions (Doublet et al., 2014; Gregorc et al., 2016; Grupe and Quandt, 2020; Harwood and Dolezal, 2020; Pettis et al., 2012), similar colony-level effects remain unclear (Collison et al., 2016). Lastly, it is well known that management of honey bee colonies by beekeepers can not only limit natural selction, but may also impose stress itself by exacerbating parasite populations and disease transmission (Neumann and Blacquière, 2016), adding to the complexity of understanding combined pathogen-parasite interactions at the honey bee colony level (O'Neal et al., 2018). Ultimately, while there are significantly more studies investigating the interactions between pesticides and pathogens on managed honey bee health, we still face various uncertainties as to what role these two stressors and their interactive effects play in understanding increased colony losses and wild honey bee health. Furthermore, findings from managed honey bee studies are most likely not ideal for predicting potential effects on wild bees (Wood et al., 2020), in particular solitary bee species, as we discuss below in more depth.

Focusing on interaction studies in wild bees, and in particular those of interactions between agrochemicals and parasites and pathogens, these are also limited in breadth. As our results and previous studies show (e.g., Siviter et al., 2021a), most of these experiments have used viruses and *Nosema* spp. In honey bees (e.g., Doublet et al., 2014; Harwood and Dolezal, 2020; Paris et al., 2020, 2018; Retschnig et al., 2015; Vidau et al., 2011), and *Crithidia bombi* in bumble bees (Baron et al., 2014, 2017; Fauser-Misslin et al., 2014; Fauser et al., 2017; Straw and Brown, 2021a). While important parasites, these are only a tiny subset of the parasites and pathogens known to infect these two groups of social bees (Schmid-Hempel, 1998). In addition, while these parasites can have significant impacts on bee health, their use in interaction experiments has also likely been driven by the presence of standard protocols for their use and the proportion of the research community who already work on them. Again, there is a general lack of knowledge of the parasite community for most of the ∼20,400 species of wild bees and their actual impact in the field. In an ideal world, interactive stressor studies would use parasites and pathogens that are known to have significant impacts on wild bees in the laboratory. In the wild, parasite impacts are driven by a combination of virulence and prevalence – highly prevalent parasites with low virulence could still overall have a higher population impact than rarely present parasites with high virulence. For example, from prevalence studies we know that *Crithidia bombi* is highly prevalent in wild bumble bees (e.g., Shykoff and Schmid-Hempel, 1991), and laboratory experiments have shown that it can have significant impacts on bumble bee health under stressful conditions (Brown et al., 2000, 2003; Yourth et al., 2008), but whether it actually impacts the population health of bumble bees in the wild remains unknown. While concerns have been raised that viral spillover from managed honey bees into wild bees might drive wild bee decline (e.g., Fürst et al., 2014), leading to the use of these viruses in wild bee studies (Meeus et al., 2014; Morfin et al., 2019; Tehel et al., 2020), we currently have no understanding of whether so-called honey bee viruses have any impact on wild bees in the field, and some studies even suggest that viruses which have previously been categorized as honey bee viruses are actually endemic in wild bee species (Manley et al., 2020; Mcmahon et al., 2015; Wang et al., 2018). Moving forward, a key need is first to identify the parasite and pathogen community of wild bees (outside of bumble bees, where it is well-known (Schmid-Hempel, 1998)). This must go beyond just detection and should include parasite and pathogen proliferation, development of disease aka clinical symptoms, and ultimately the impact of these organisms on the fitness of a given host, host colony and possibly entire populations. This will enable us to determine which of these parasites and pathogens actually have meaningful impacts on wild bee population health. Only then can we make sensible choices of which parasites to use in interactive experiments with agrochemicals.

Most studies of interactions between stressors in bees have involved insecticides (Siviter et al., 2021a). This focus has arguably been driven by the production, marketing, agricultural application, and scientific investigation of neonicotinoids, a group of systemic insecticides. Initial high profile studies of the impact of neonicotinoids on bee health (e.g., Gill et al., 2012; Henry et al., 2012; Whitehorn et al., 2012) led to both an explosion of research and huge public engagement, which fed on each other to produce a scientific industry of examining all aspects of these insecticides on bee health. While this resulted in the banning of three neonicotinoids for outside use in the EU, these insecticides are still widely used around the world, and research into their impact continues. At the same time, new insecticides have been introduced and examined for their possible impacts (Brown et al., 2016; Siviter et al., 2018; Siviter and Muth, 2020). With the recognition that interactions between stressors might play a key role in reducing bee health (e.g., Vanbergen et al., 2013), it is perhaps no surprise that most interaction studies have included insecticides as one of the stressors.

However, insecticides are not the only agrochemical group that could impact bee health. Herbicides and fungicides are heavily used around the globe, and have been shown to have negative effects on bee health (e.g., Belsky and Joshi, 2020), as have other ingredients within agrochemical applications (e.g., Straw and Brown, 2021a). Given this, studies of interactions between stressors need to incorporate a more balanced approach, which recognizes the potential importance of other agrochemicals (Straw et al., 2022). This, in turn, requires a knowledge of the extent to which wild bees are exposed to these other agrochemical stressors, as without this, experiments cannot assess real-world hazard or risk (Mesnage et al., 2021; Straw et al., 2022). Of the ∼20,400 species of wild bees, actual exposure to any agrochemical has only been investigated for a handful of species (mainly from the genera *Bombus*, *Osmia*, *Megachile*, or *Melipona*), and this is a major lacuna that urgently needs to be filled.

Indeed, our review revealed a striking lack of empirical data from designed experiments to examine interactive effects of pesticides and pathogens in solitary wild bees. All studies on wild bees found in our systematic literature search have been conducted on social bees, focusing on only two pathogen taxa (*Crithidia* and *Nosema* spp.). However, the vast majority of the more than 20,000 species of wild bees worldwide (approximately 70% in temperate biogeographic regions) are solitary (Engel et al., 2020; Michener, 2000). A solitary life form implies that a female bee constructs her nest and provisions offspring alone, without cooperation with conspecifics. As a consequence, adverse effects of pesticides, pathogens and their interactions should have more pronounced impacts on solitary bee populations compared to social bees, because negative effects e.g. on mortality or performance of nesting females will directly impair fitness, while social bees should be able to buffer negative impacts to some extent at the colony level (Sgolastra et al., 2019; Straub et al., 2015). Thus, there is an urgent need to extend studies on the impact of pesticides, pathogens and their interactions on a range of solitary bee species, but also wild honey bees and other social bee species (e.g., stingless bees).

This plea is underpinned by increasing evidence that the levels and pathways of exposure to individual and combined stressors, as well as a bee's sensitivity to them, strongly depends on specific life-history traits (Arena and Sgolastra, 2014; Brittain and Potts, 2011; Grozinger and Flenniken, 2019; Kopit et al., 2021; Kopit and Pitts-Singer, 2018; Proesmans et al., 2021; Truitt et al., 2016; Uhl et al., 2016). For example, solitary and social bee species differ in activity and nesting period, nesting duration, voltinism, body size, foraging range, habitat preference, food plant preference and level of diet specialization, the level of pollen and nectar consumption as adults and larvae, as well as their mode of nesting (i.e., ground-nesting in the soil or above-ground nesting using different nesting structures) and use of nesting materials (e.g., mud, leaves, plant pubescence), which likely results in different routes and levels of exposure to different pesticide contamination and pathogen infection routes (Proesmans et al., 2021; Sgolastra et al., 2019; Uhl and Brühl, 2019). A large knowledge gap concerns the potential exposure of ground-nesting solitary bees to pesticides accumulating in soils, e.g. as adult female bees excavating soil material to construct nests, or as developing larvae through contact with soil that forms nest cells, although a water-resistant coating applied to nest cells may reduce this exposure risk in many ground-nesting species (Chan et al., 2019). Furthermore, a bee species' sensitivity towards different pesticides can vary strongly between social and solitary bee species (Sgolastra et al., 2020; Wood et al., 2020). Body size can be an important trait affecting such sensitivity (Arena and Sgolastra, 2014; Uhl et al., 2016). Social and solitary bee species also vary in different aspects of physiology, e.g., the detoxification abilities and pathways of different taxa (Hayward et al., 2019).

Similarly, species-specific traits likely play an important role in governing inter- and intraspecific transmission of pathogens and a wild bee species’ infection risk (Graystock et al., 2016; Manley et al., 2015; Proesmans et al., 2021). For instance, foraging traits of bees, such as diet breadth and preference, along with plant and pathogen traits are likely drivers of horizontal transmission of pathogens between different bee species sharing flowers in plant-bee-pathogen interaction networks (e.g., Figueroa et al., 2020; Graystock et al., 2020, 2016; McArt et al., 2014; Proesmans et al., 2021). Sociality is a further key trait affecting pathogen exposure, transmission, and resistance (Cremer et al., 2007). For example, cooperative brood care, along with overlapping generations in densely populated colonies facilitate disease spread in colonies of social bees (Cremer et al., 2007; Graystock et al., 2015; Manley et al., 2015). Further, the typically generalised floral diets and long colony cycles of most social wild bees contribute to increased direct and indirect (e.g., via shared flowers) contact with other bees and thus pathogen infection risk (Proesmans et al., 2021). However, social bee species have also developed mitigation strategies to reduce risks of high pathogen loads through social immunity (i.e., behavioural, physiological and organisational adaptations of the colony level to prevent pathogen entrance, establishment, and spread (e.g., Cremer et al., 2007; Meunier, 2015; Wilson-Rich et al., 2009a). While social immunity in bees has received relatively high attention, we have little understanding of the biological mechanisms behind it, which may be impaired by pesticide exposure, and even less is known about how pesticides may reduce individual immunocompetence (e.g., reduced induction of antimicrobial peptides or haemocyte production (Brandt et al., 2020; Collison et al., 2016)) and increase pathogen infestation and pathogen loads in solitary bees, and to what extent such mechanisms may vary among species of different phylogenies and traits (Brandt et al., 2020).

Moreover, pathogen research is heavily biased towards social bees, and our knowledge on pathogen and parasite communities in solitary wild bees is scarce (Tehel et al., 2016). Although there is increasing evidence for single-stranded RNA viruses or *Crithidia* ssp. Crossing phylogenetic boundaries, and therefore possibly being present in a range of different solitary bee taxa (Mcmahon et al., 2015; Ravoet et al., 2014), there is less evidence that these pathogens are also able to replicate in such solitary bee hosts (e.g., Radzevičiūtė et al., 2017; V. Strobl et al., 2019; Tapia-González et al., 2019), and whether they frequently adversely affect fitness and populations dynamics of solitary bees remains unclear (Dolezal et al., 2016; Tehel et al., 2016, 2020). It also remains unknown, whether potential negative effects of pathogens may be additively or synergistically reinforced by pesticides under field conditions (Brandt et al., 2020; Collison et al., 2016).

All identified studies that have addressed interactive effects of pesticides and pathogens on wild bees using a crossed design have been conducted under laboratory conditions. Laboratory studies have clear advantages such as (i) the ability to control for a variety of confounding factors potentially affecting measured response variables in addition to applied treatments, (ii) the availability of well-established and repeatable protocols, (iii) no logistical constraints to achieve – depending on the tested factors and study system – sufficient replication and low risk of type II statistical errors (i.e., a real effect of a tested explanatory variable is not detected due to insufficient experimental replication). Laboratory studies are therefore highly suitable to precisely estimate effect sizes of single and combined treatment factors under study, to provide proof of concepts and test hypotheses on interactive effects, and to draw conclusions about mechanistic relationships of interactive effects of specific pesticides and pathogens (Medrzycki et al., 2013). Hence, such laboratory assessments using standard protocols have traditionally been the cornerstone of regulatory risk assessments processes (e.g., EFSA, 2014a; OECD, 1998). However, the advantages of reducing complexity and excluding various influencing factors characterizing real-world systems come at a high price. Ignoring them may lead to unrealistic estimates of effect sizes and potentially wrong conclusions about the existence and magnitude of impacts of pesticide-pathogen interactions on wild bees (Sgolastra et al., 2020; Topping et al., 2021; Van Oystaeyen et al., 2020).

Among the many pitfalls of laboratory experiments in addressing pesticide-pathogen interactions are importantly unrealistic or irrelevant concentrations of pesticides used (Carreck and Ratnieks, 2014). To avoid this pitfall, knowledge of the extent to which wild bees are exposed to the studied pesticide(s) is essential (Mesnage et al., 2021; Sanchez-bayo and Goka, 2014). However, such data are currently largely lacking for many pesticides and exposure scenarios for different wild bee species (Kopit and Pitts-Singer, 2018; Main et al., 2020). But experiments cannot assess real-world risks of pesticides and their interactive effects with pathogens on wild bees without such data (EFSA, 2014b; Mesnage et al., 2021; Sgolastra et al., 2020, 2019). Similarly, laboratory studies testing effects and underlying mechanisms of interactions between pesticides and pathogens should ensure realistic infection scenarios and pathogen loads for different wild bee species. Yet, our understanding of infection pathways and pathogen loads is very limited for most pathogens and wild bee taxa, which is particularly true for solitary bees (IPBES, 2016; Tehel et al., 2016). Here, field studies are of critical importance to identify which pathogens are actually relevant for which wild bee taxa to address this fundamental knowledge gap (Dicks et al., 2021). Furthermore, ignoring important co-drivers of bee health such as interactions with further key stressors such as nutritional stress (Stuligross and Williams, 2020) may lead to under- or overestimating impacts of pesticides, pathogens, and their interactions on wild bee health (Carreck and Ratnieks, 2014; Goulson et al., 2015b; Siviter et al., 2021a; Topping et al., 2021; Vanbergen et al., 2013).

Furthermore, certain key response variables can only be reliably studied under (semi-)field conditions. For example, measuring effects of pesticides, pathogens and their interactions on reproductive success and fitness is crucial to understanding their impacts on populations of wild bees and their long-term trends (IPBES, 2016; Straub et al., 2020). This requires experimental settings in which wild bees can nest, forage and provision their offspring ideally during their entire life cycle, which typically need to be (semi-)field settings (Sgolastra et al., 2020; Van Oystaeyen et al., 2020). To move forward, we therefore urgently need more studies of pesticide-pathogen interactions under field-realistic conditions. However, field studies addressing pesticide-pathogen interactions are challenging in many respects. The less controlled and more complex and variable systems are, and the smaller effect sizes of treatments to be detected, the greater the need for high replication to detect such effects reliably (Cresswell, 2011; EFSA, 2013; Woodcock et al., 2016a). In addition, (semi-)field studies are typically conducted at much larger spatial and temporal scales to adequately embrace natural behaviours and life cycles of wild bees, and they are therefore labour, time, and cost-intensive. It can also be challenging to reproduce field studies across different environmental systems varying in a range of influencing factors such as climatic conditions, land use types etc. (e.g., Woodcock et al., 2017). A key challenge of field experiments studying interactive effects of pesticides and pathogens on wild bees is therefore to balance the level of control and complexity (Suryanarayanan, 2015). More “control-orientated” study designs risk failing to adequately account for important indirect and multifactorial processes affecting bee health, while more “complexity-orientated” studies have a higher risk to fail to detect significant effects and mechanistic relationships for factors of interest (type II statistical error, see above (Woodcock et al., 2016a)).

In addition, the deliberate use and potential spread of pathogens for research purposes in field experiments poses significant ethical concerns. Field studies therefore generally rely on quantifying existing pathogen prevalence and loads in wild bees (e.g., Wintermantel et al., 2018). However, researchers can perform *a priori* pathogen screenings and use this information to design experiments testing for single and combined impacts of pathogens and pesticides to increase control over these factors in field experiments and integrate mechanistic models as guidance to design experiments for relevant pesticide-pathogen interactions (Campbell et al., 2016). A promising first step moving forward towards more field-realistic studies on pesticides and their interactions with pathogens or further stressors of wild bees are semi-field experiments (Bramke et al., 2019; Stuligross and Williams, 2020). Such experiments ideally combine advantages such as field-realistic exposure routes and levels, long-term assessments and include measures of fitness and population growth with those of a high level of control of influencing factors e.g. by using caged wild bee populations or colonies (e.g., Strobl et al., 2021a; Strobl et al., 2021b; Tamburini et al., 2021). Last but not least, risks of pathogen spread, at least for some pathogen groups, can be minimized in semi-field experiments conducted with caged wild bees (e.g., Bramke et al., 2019).

If we aim to understand pesticide-pathogen interactions, and so effectively mitigate their role in the ongoing loss of wild bee species, we must first strive to improve our understanding of how these stressors individually act on bees. To do so, it appears essential to take evolutionary biology into account and we, therefore, propose future studies should have a stronger focus on fitness, the essential factor governing all wild populations (Straub et al., 2020). For instance, studies using PCR and qPCR methods to detect the prevalence of certain pathogens (e.g., viruses) in bees in the field do not provide proof of an infection (Brown, 2017). As pathogens are likely to be encountered on shared, contaminated food resources (i.e., flowers), the detected pathogen may not even be in the bee, but rather only on the surface of the body. In addition, the bee may only act as a transient host without causing infection or any pathogenic effects to the host (Durrer and Schmid-Hempel, 1995). To exclude these possibilities and adequately address the role of pathogens on wild bee populations, studies must first provide robust evidence that an infection is indeed occurring and that there are clear fitness constraints (e.g., fewer offspring produced) or at least on fitness-relevant traits (e.g., male sperm capacities). The same holds true for understanding the role of pesticide exposure on wild bee declines. While a plethora of studies have demonstrated negative impacts of various pesticides on bees and other pollinators (Blacquière et al., 2012; Lu et al., 2020), clear knowledge gaps remain as to how pesticides affect wild bee populations under field conditions. Despite previous large-scale field studies showing causal data suggesting reduced wild bee density and population growth due to pesticide exposure (Rundlöf et al., 2015), the underlying mechanisms are yet to be identified. As it is close to impossible to test each and every pesticide and pathogen interaction in each of the ∼20,400 bee species, we must strive to improve our understanding of the underlying mechanistic pathways and how frequent they are across different phylogenetic groups of bees. Only then can we use this knowledge to design more relevant experiments in terms of involved pesticides, pathogens, and bee species, and also move towards a more predictive science and modelling of interactive effects in different environmental contexts (e.g., Topping et al., 2021). Thus, there is an urgent need for additional long-term data on the likelihood of pesticide and pathogen exposure of wild bee communities in the field, as well as data revealing direct causality between such exposure and the loss of wild bee abundance and richness (Brühl et al., 2021; Rundlöf et al., 2015).

## 5 Conclusions

There is a clear need for empirical field studies on a range of pesticides, pathogens, and wild bee species to better understand the nature of interactions, underlying mechanisms, and in particular their relevance for bee fitness. Based on our review it is currently not possible to draw general conclusions on the role of pesticide-pathogen interactions in the ongoing decline of wild bees. However, it appears clear that the interaction of these stressors must be considered within context. Indeed, Bird et al. (2021) revealed that at least for managed honey bees, pesticide-pathogen interactions often yielded antagonism and that the common assumption of additive or synergistic effects may be overrated. For wild bees, habitat destruction and degradation, and the subsequent side effects (e.g., loss of adequate floral food resources, nesting sites or increased fragmentation of food and nesting habitats and thus longer foraging distances (Ganser et al., 2021)) in combination with the ongoing threat of climate change are likely to be far more profound factors (Brown and Paxton, 2009; Dicks et al., 2021; IPBES, 2016). A holistic approach is therefore required to first identify the most common and most severe stressor interactions (i.e., synergism) in the natural habitats of various bee species. Later, standardized laboratory studies can help improve our understanding of the physiological and genetic mechanisms underlying such interactions and how they negatively affect fitness. In a final step, these findings must be investigated under field conditions to provide reliable data for models to predict the interactive effects of stressors and so protect bees and other insect species from future risks. Furthermore, it appears long overdue that regulatory authorities incorporate the evaluation of combined stressor interactions into current environmental risk assessments (Topping et al., 2020), including estimates of fitness as the key factor governing any wild population (Straub et al., 2020). This would not only improve our understanding of how stressors interact but also reflect a more field-realistic scenario and enable policy-makers to implement adequate and sustainable measures to safeguard biodiversity.

## Funding

This project received funding from the European Horizon 2020 research and innovation programme under grant agreement no. 773921. Funding was provided by the Vinetum Foundation to L.S., V.S., O.Y., and P.N and the Swiss Federal Office for the Environment to L.S. and P.N.

## Conflicts of interest

The authors declare they have no conflicting interests.
